# Interstitial lung disease exacerbations following initiation of nerandomilast: A case series

**DOI:** 10.1016/j.rmcr.2026.102512

**Published:** 2026-09-29

**Authors:** Traci N. Adams

**Affiliations:** University of Texas Southwestern Medical Center, Division of Pulmonary and Critical Care, Medicine, USA

## Abstract

Nerandomilast is a novel PDE4 inhibitor approved for the treatment of idiopathic pulmonary fibrosis (IPF) and progressive pulmonary fibrosis (PPF). This case series reports 5 patients with an acute exacerbation of IPF or PPF within 2 weeks of administration of nerandomilast. One patient was on background nintedanib and none were on pirfenidone at the time of the exacerbation. All exacerbations were confirmed by a change in dyspnea and/or cough, worsening oxygen requirement, and new ground glass infiltrates on CT. All required hospitalization. A suggested mechanism for exacerbations on nerandomilast includes upregulation of cAMP by nerandomilast, which can drive IL-17 production. IL-17 has a known association with IPF exacerbations. The results of this case series need to be evaluated prospectively and ongoing pharmacovigilance is required.

## 1 Introduction

Idiopathic pulmonary fibrosis (IPF) and progressive pulmonary fibrosis (PPF) are interstitial lung diseases (ILD) with a progressive course [1]. Existing therapies for IPF and PPF, including pirfenidone and nintedanib, slow the rate of decline of forced vital capacity (FVC) in these conditions [[2], [3], [4]], but the majority of patients continue to decline on therapy, with the only curative treatment being lung transplant [[1], [2], [3], [4]]. In addition, both pirfenidone and nintedanib have a substantial side effect profile [[2], [3], [4]]; thus, new treatments to slow or halt progression in IPF and PPF with a lower burden of side effects are needed.

Nerandomilast is phosphodiesterase 4 (PDE4) inhibitor recently approved for the treatment of IPF and PPF [5,6]. Clinical trials demonstrated that nerandomilast slowed decline in FVC compared to placebo with no change in the incidence of IPF or PPF exacerbations in patients on nerandomilast compared to placebo [5,6].

Post-marketing surveillance is an important part of monitoring newly approved drugs, as side effects may be seen after drug approval due to the increased number of patients on the medication. This case series reports 5 patients with an exacerbation of ILD following initiation of nerandomilast.

## 2 Methods

Four patients with an acute exacerbation of IPF or PPF after starting nerandomilast were identified retrospectively in the UTSW ILD clinic. Clinical characteristics, including the type of ILD, treatment history, pulmonary function testing, computed tomography (CT) images, and hospital courses during exacerbation were recorded.

An exacerbation of ILD is considered a life-threatening event and is defined as an acute, clinically significant respiratory deterioration characterized by evidence of new widespread alveolar abnormality in a patient with a previous or concurrent diagnosis of IPF or PPF, acute worsening of dyspnea typically < 1 month in duration, new bilateral ground glass abnormality superimposed on a background of ILD, and not fully explained by heart failure or fluid overload [7].

### 2.1 Ethics statement

This study was conducted in accordance with the amended Declaration of Helsinki and was considered exempt by the University of Texas Southwestern Medical Center (UTSW) Institutional Review Board (STU20261619). Consent was not required due to the small number of patients included and the use of de-identified data only. Ethnicity was self-reported by study subjects in the medical record, and subjects were not stratified or analyzed on the basis of confounding variables such as socioeconomic status or nutrition; this information was not available in the medical record and was not included. Data for this study was collected between August 9, 2026-August 11, 2026, though the patients included were treated between November 2025 when nerandomilast was first approved through August 11, 2026. Identifying data was not recorded or accessed during or after data collection.

Case 1 Patient 1 is a 66 years old man with fibrotic hypersensitivity pneumonitis (HP). The diagnosis of HP is based on mold exposure in the home and a typical HP pattern on high resolution computed tomography (HRCT) of the chest. His past medical history is notable for coronary artery disease, hypertension, and hemochromatosis. He developed progressive pulmonary fibrosis on the basis of > 10% decline in forced vital capacity (FVC), increased oxygen requirement from 3.5 LPM to 10LPM with exertion despite removal from exposure. He was first initiated on pirfenidone by an outside pulmonologist for < 6 months, as he did not tolerate the medication due to gastrointestinal side effects. He then transferred his care to UTSW ILD clinic and was initiated on mycophenolate 1000mg twice daily as well as nintedanib for PPF, but he stopped nintedanib due to development of jaundice. He was referred to evaluation for lung transplant. Three months after discontinuation of nintedanib, he was initiated on nerandomilast 18mg twice daily.

Two weeks after initiating nerandomilast he presented to lung transplant clinic with increased shortness of breath for the past 1 week, pursed lip breathing on exam, and an increase in oxygen requirement confirmed on 6 minute walk test from 4LPM at rest and 10LPM with exertion 2 months prior to 8LPM at rest and 15LPM with exertion and a decline in 6 minute walk distance from 189m to 154m. He was admitted to the hospital from transplant clinic. On admission, he was afebrile without leukocytosis, and on exam he had fine crackles in the lungs and no lower extremity edema (Table 1Table 1). Respiratory viral panel was negative. CT chest on admission revealed an increase in both degree of fibrosis and ground glass opacities compared to CT done 1 year prior (Fig. 1A, Fig. 1BA and B). He was initiated on a prednisone taper.

**Table 1:** Physical examination findings and laboratory values on presentation for ILD exacerbation.

	Patient 1	Patient 2	Patient 3	Patient 4	Patient 5
Temperature (°C)	36.6	37.0	36.7	36.6	36.1
Lower extremity edema (Yes/No)	No	Yes	No	No	No
Lung auscultation findings	Coarse breath sounds bilaterally	Tachypnea, bibasilar crackles	Fine crackles in lower half of lung fields	Coarse breath sounds	Tachypnea, rales
Jugular venous distension (Yes/No)	No	No	No	No	No
Nt-pro BNP pg/mL	65	1719 (baseline 1713 6 months prior)	Not done	69	113
White blood cell count (x10(9)/L)	10.22	10.01	Not done	15.31 (baseline 12.26)	11.9
Neutrophils (x10(9)/L)	9.73	5.78		9.05	9.44
Immature granulocytes (x10(9)/L)	0.03	0.20		0.25	0.04
Respiratory viral panel result	Negative	Negative	Not done	Negative	Negative
Sputum culture result	Not done	Negative	Not done	Not done	Negative
Echocardiogram inferior vena cava (IVC) caliber and collapsibility	Normal IVC caliber and collapsibility	Normal IVC caliber and collapsibility	Not done	Normal IVC caliber and collapsibility	Normal IVC caliber and collapsibility

**Fig. 1A:**
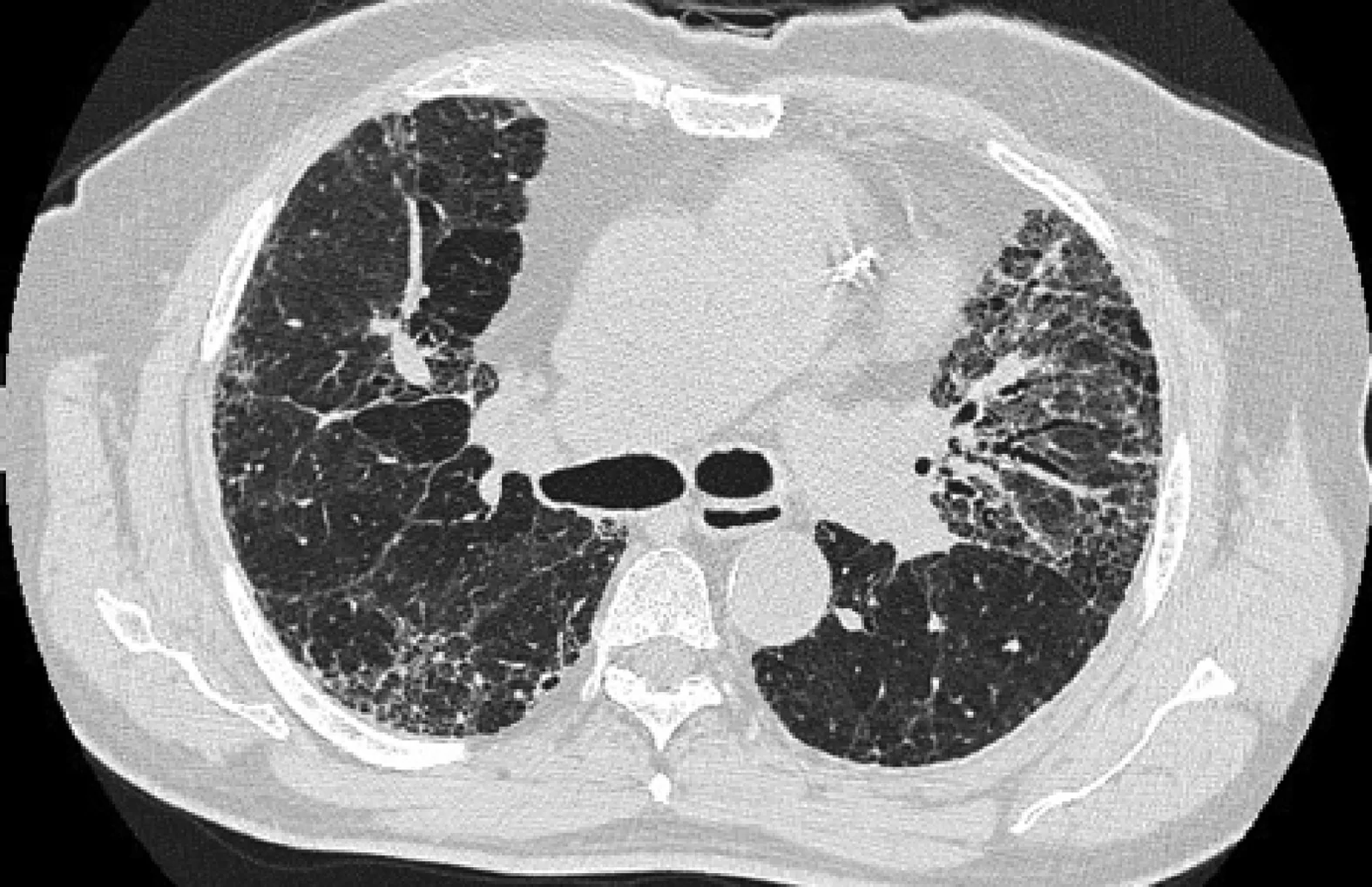
Patient 1 CT 1 year prior to initiation of nerandomilast.

**Fig. 1B:**
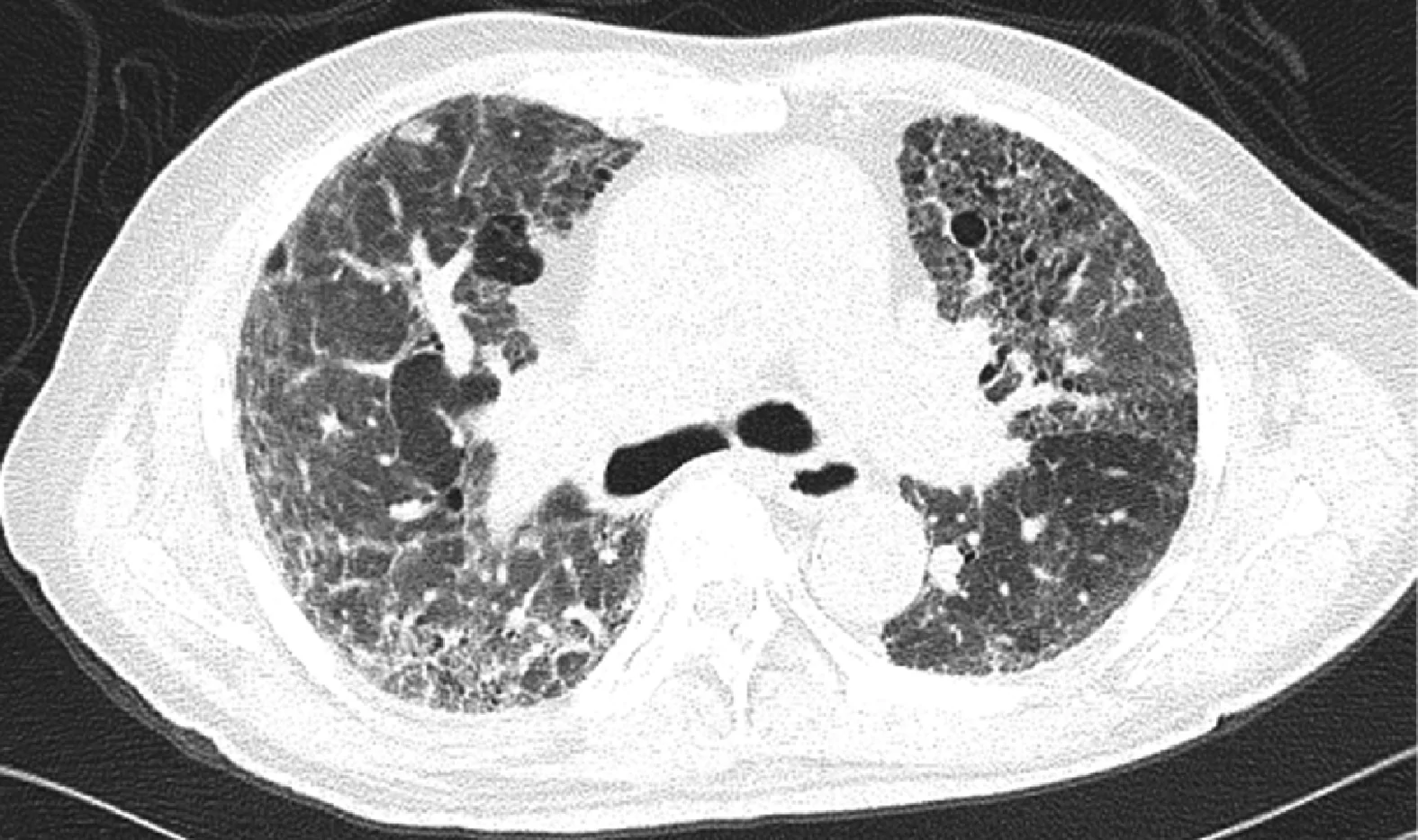
Patient 1 CT on admission for ILD exacerbation.

One month after discharge, his resting oxygen requirement had declined to 6LPM, though he remained on 15LPM with exertion. His FVC improved from 1.85L at the time of the exacerbation to 2.13L. The patient was declined for lung transplant due to comorbidities. Mycophenolate 1000mg twice daily and nerandomilast were continued and he has had no further exacerbations in the 2 months since his admission.

Case 2 Patient 2 is a 78 year old female with a history of pulmonary hypertension on tadalafil and macitentant, atrial fibrillation, prior left breast cancer, atrial fibrillation, extranodal histiocytosis, and ILD. Her ILD had been detected 5 years prior by HRCT, but at the time of referral to UTSW ILD clinic, she was not on any therapy and had an FVC of 70% predicted and a DLCO of 29% predicted. She was too ill to tolerate bronchoscopy or surgical lung biopsy. She was initiated on nerandomilast for progressive fibrotic ILD.

She developed diarrhea on nerandomilast 18mg twice daily and stopped the medication after 3 days. She was dose-reduced to 9mg, and she was admitted 2 weeks later with dyspnea, pleuritic chest pain, dry cough, and increased oxygen requirement from 4LPM to 10LPM at rest. On admission, she was afebrile without leukocytosis, and on exam she had fine crackles in the lungs and no lower extremity edema. CT chest revealed new LUL multifocal ground glass opacities compared to HRCT done 3 months prior (Fig. 2A, Fig. 2BA and B). She was treated with diuretics, antibiotics, and a 7 day course of prednisone. She was admitted again 1 month later with worsening dyspnea, and CT chest revealed new multifocal opacities (Fig. 2C). She was discharged on a 6 week prednisone taper.

**Fig. 2A:**
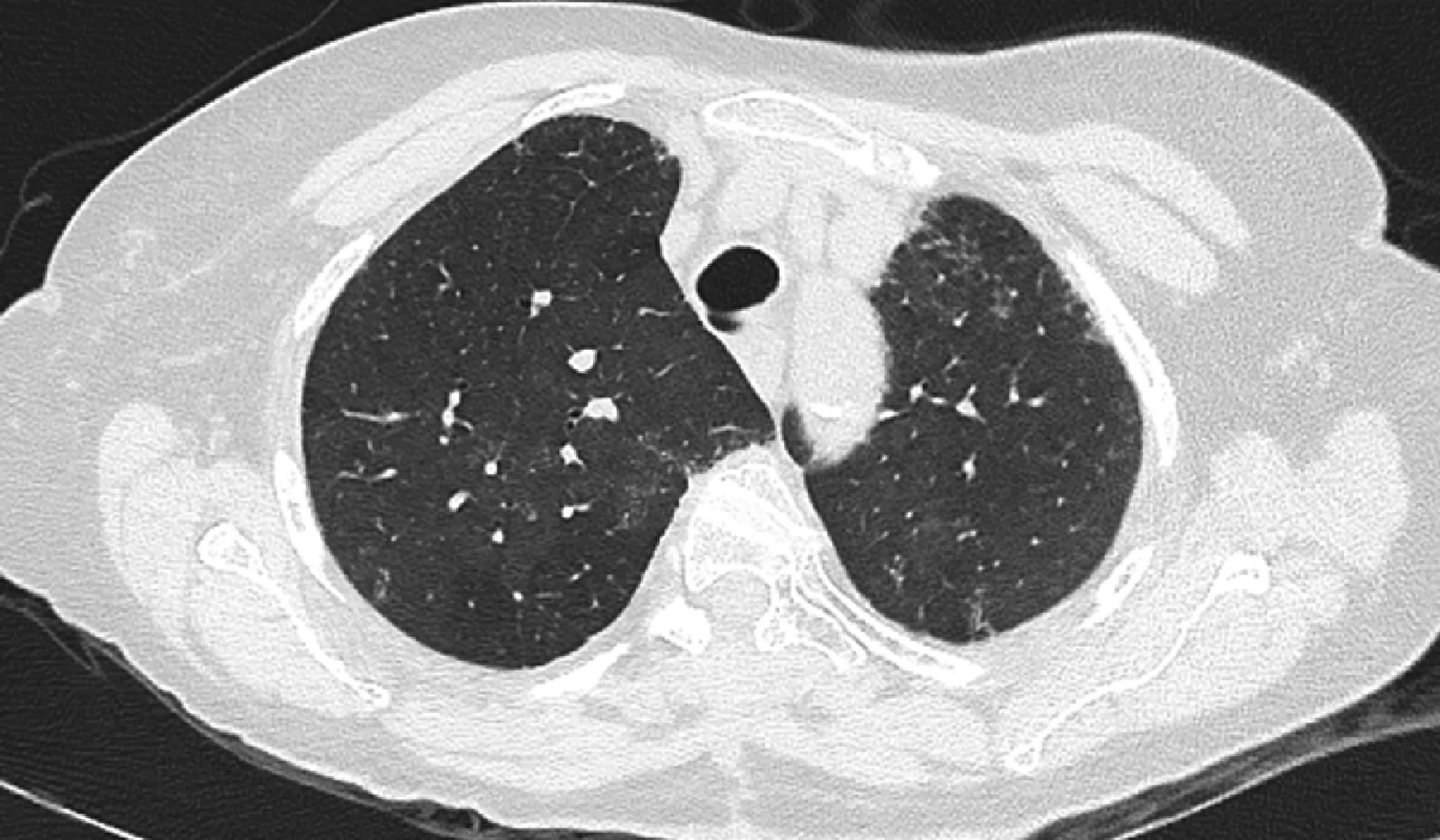
Patient 2 CT 3 months prior to initiation of nerandomilast.

**Fig. 2B:**
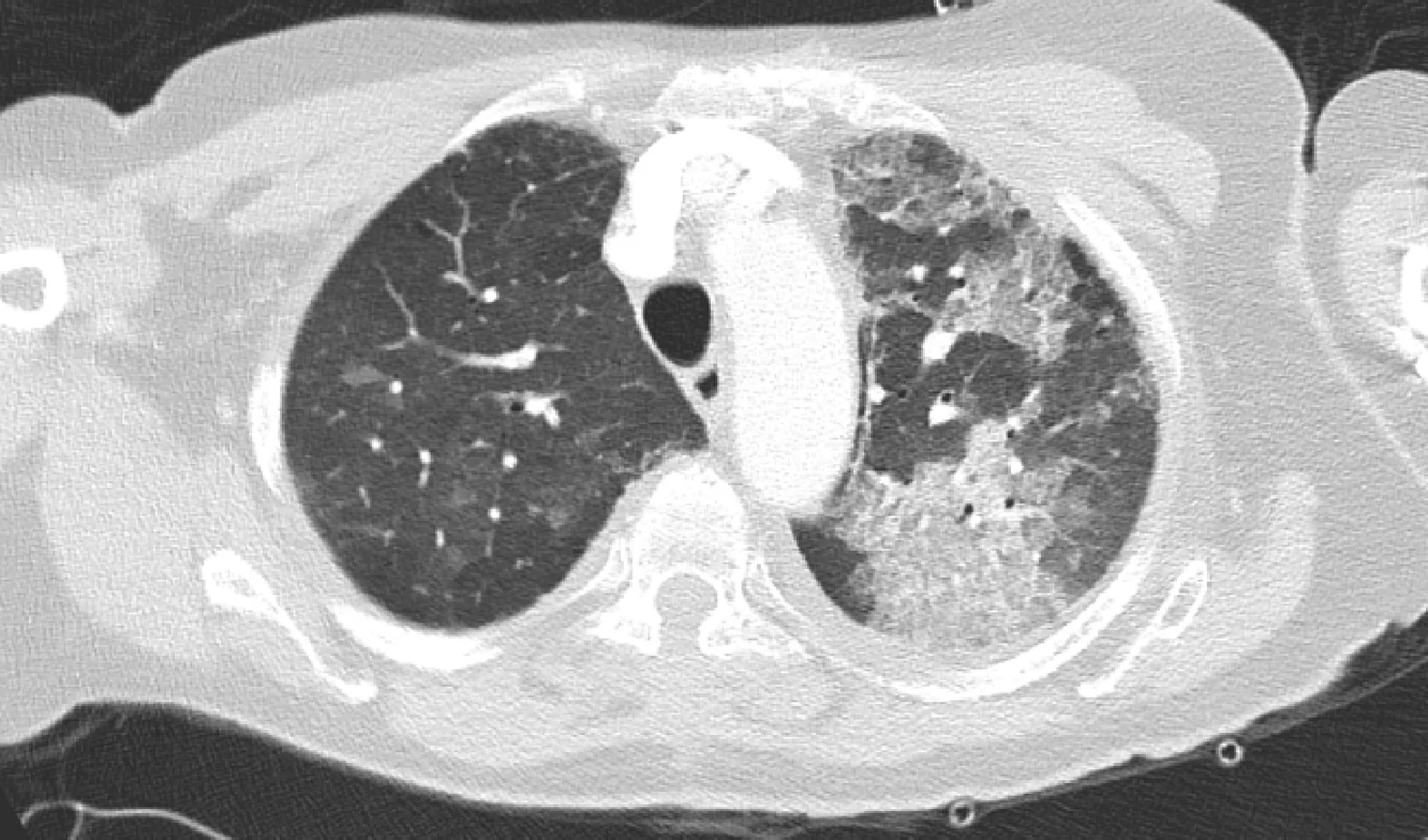
Patient 2 CT on admission for ILD exacerbation.

**Fig. 2C:**
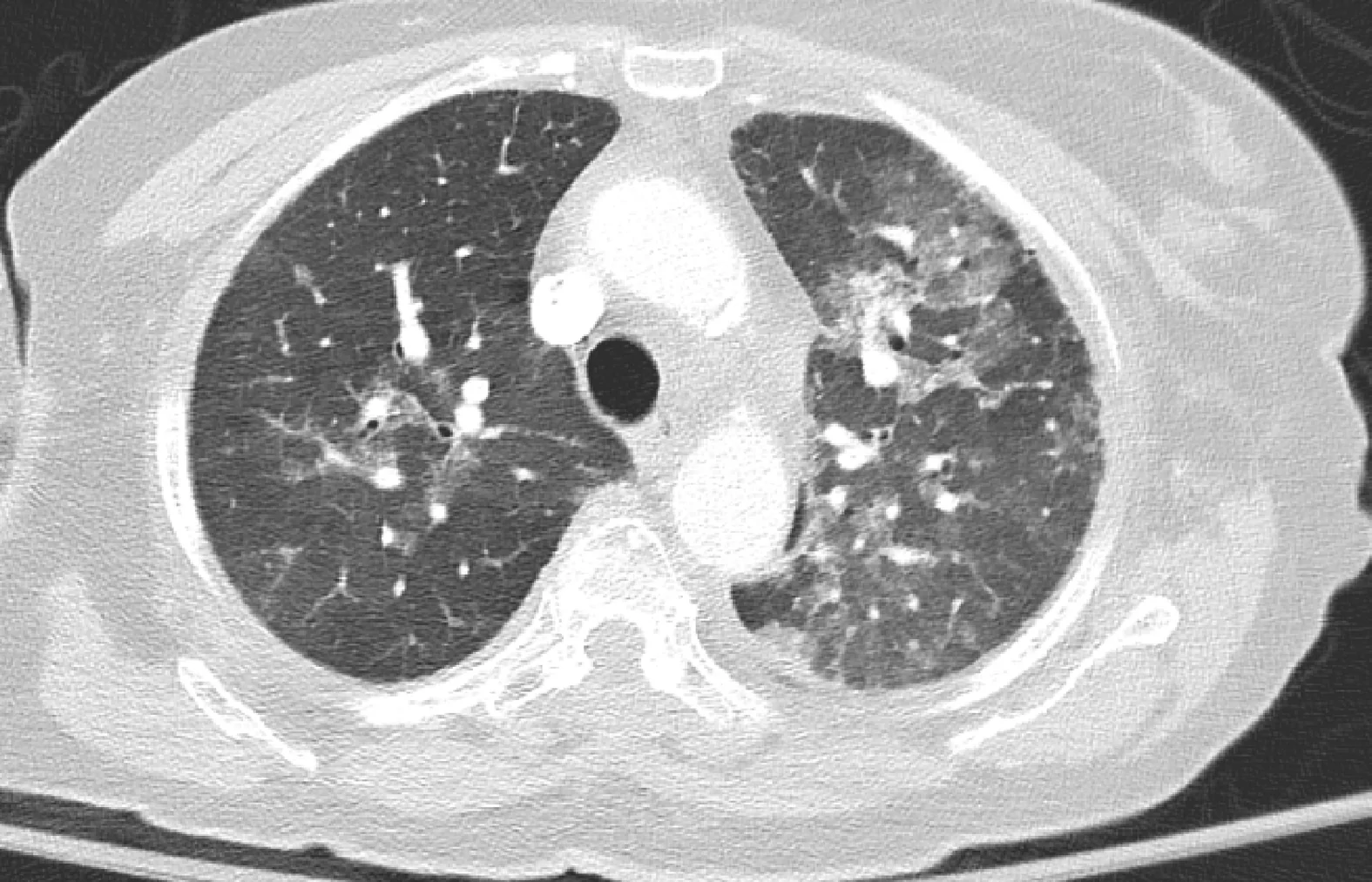
Patient 2 CT on re-admission for ILD exacerbation 1 month after initial admission.

When she returned to pulmonary clinic following treatment of the exacerbation, her FVC had declined to 56% predicted and her DLCO to 26% predicted. Her CT revealed improvement in ground glass opacities (Fig. 2D).

**Fig. 2D:**
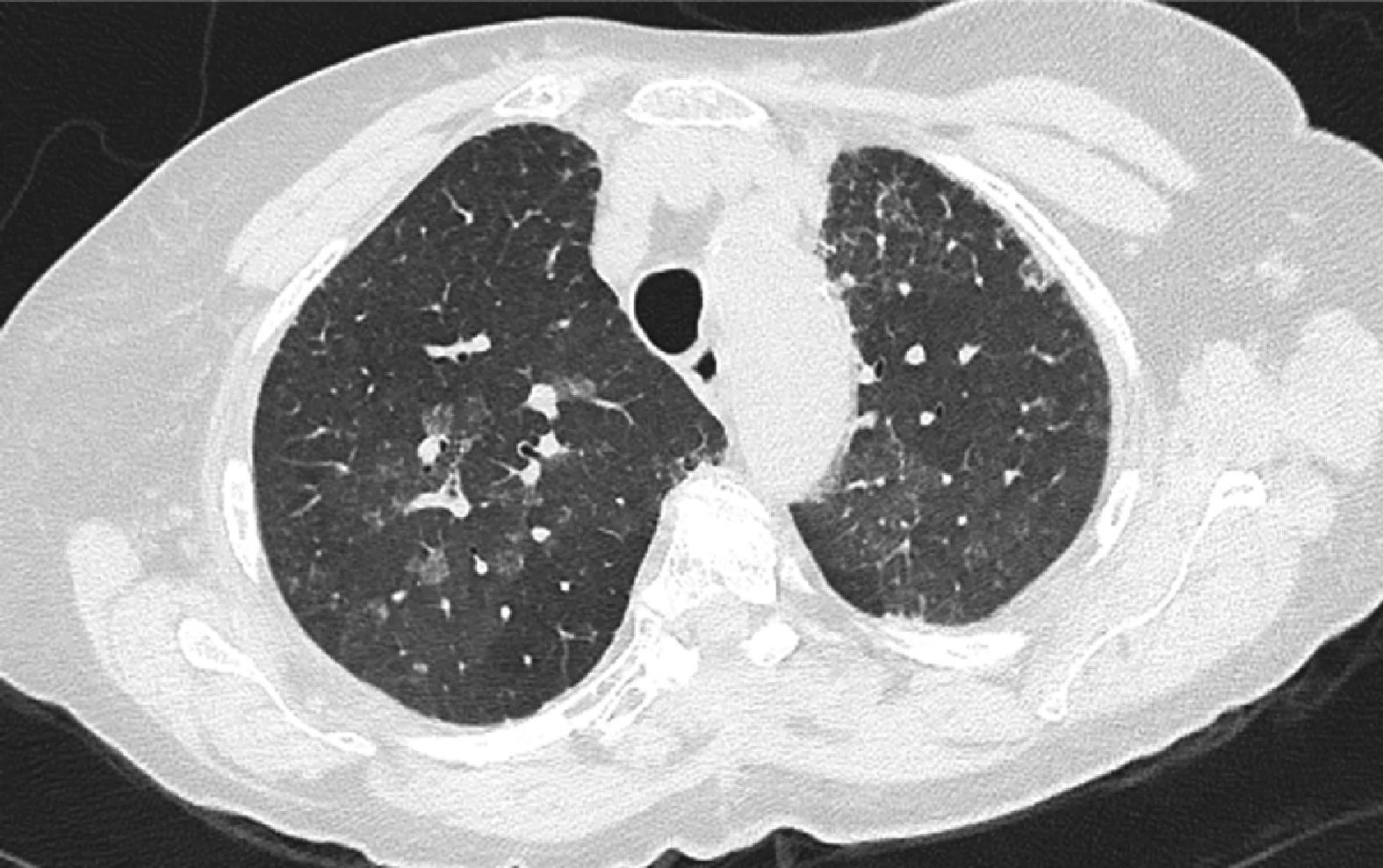
Patient 2 CT 3 months after discharge.

Case 3 Patient 3 is an 83 year old male with unclassifiable ILD. ILD was first seen on CT chest in 2021. He was diagnosed with IPF by outside pulmonologist. He was unable to tolerate nintedanib 150mg or 100mg twice daily due to intractable diarrhea. He was unable to tolerate pirfenidone at a dose of 267mg tid due to debilitating fatigue, so pirfenidone was discontinued. He established with ILD clinic at UTSW in 2024, and BAL revealed 46% lymphocytes. He was too ill to tolerate biopsy. He was trialed on mycophenolate and then azathioprine but failed to tolerate either drug. He was initiated on nerandomilast due to decline in FVC, increased fibrosis by CT, and worsening symptoms over the prior year.

Seven days after starting newranomilast 18mg twice daily, he developed worsening cough and dyspnea. 6 minute walk test revealed a new oxygen requirement of 3LPM with exertion compared to 4 months prior when he had performed a walk test on room air, and he was admitted to the hospital. On admission, he was afebrile without leukocytosis, and on exam he had fine crackles in the lungs and no lower extremity edema. CT chest revealed interval development of patchy ground glass opacities in a background of fibrotic lung disease compared to 2 months prior to initiation of nerandomilast (Fig. 3A, Fig. 3BA and B). He was treated with a 6 week prednisone taper. His lung function did not recover following the exacerbation, with his forced vital capacity declining to 1.20L at his clinic appointment 2 months after the exacerbation compared to 1.72L prior to initiation of nerandomilast. He was referred to palliative care.

**Fig. 3A:**
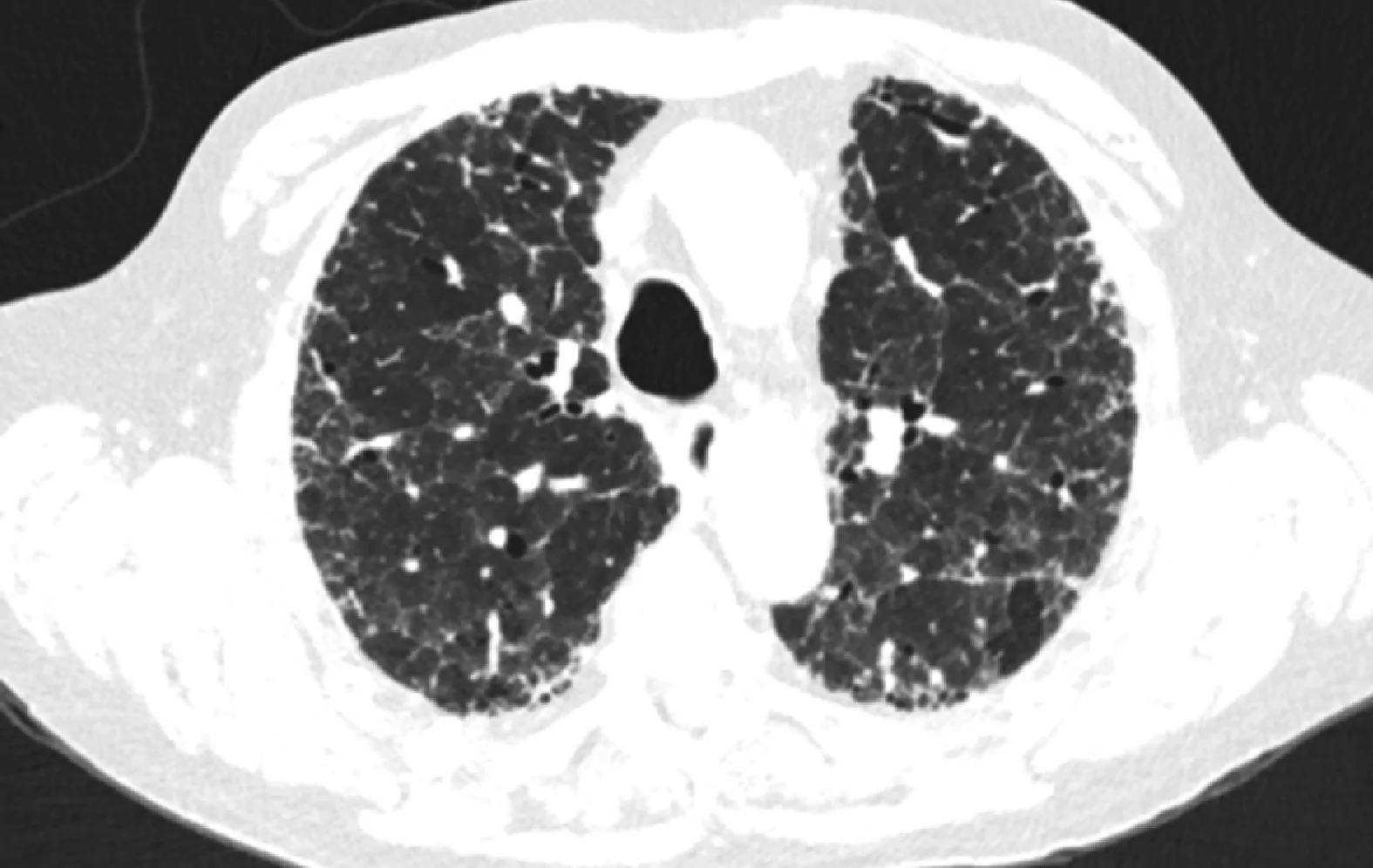
Patient 3 CT 2 months prior to initiation of nerandomilast.

**Fig. 3B:**
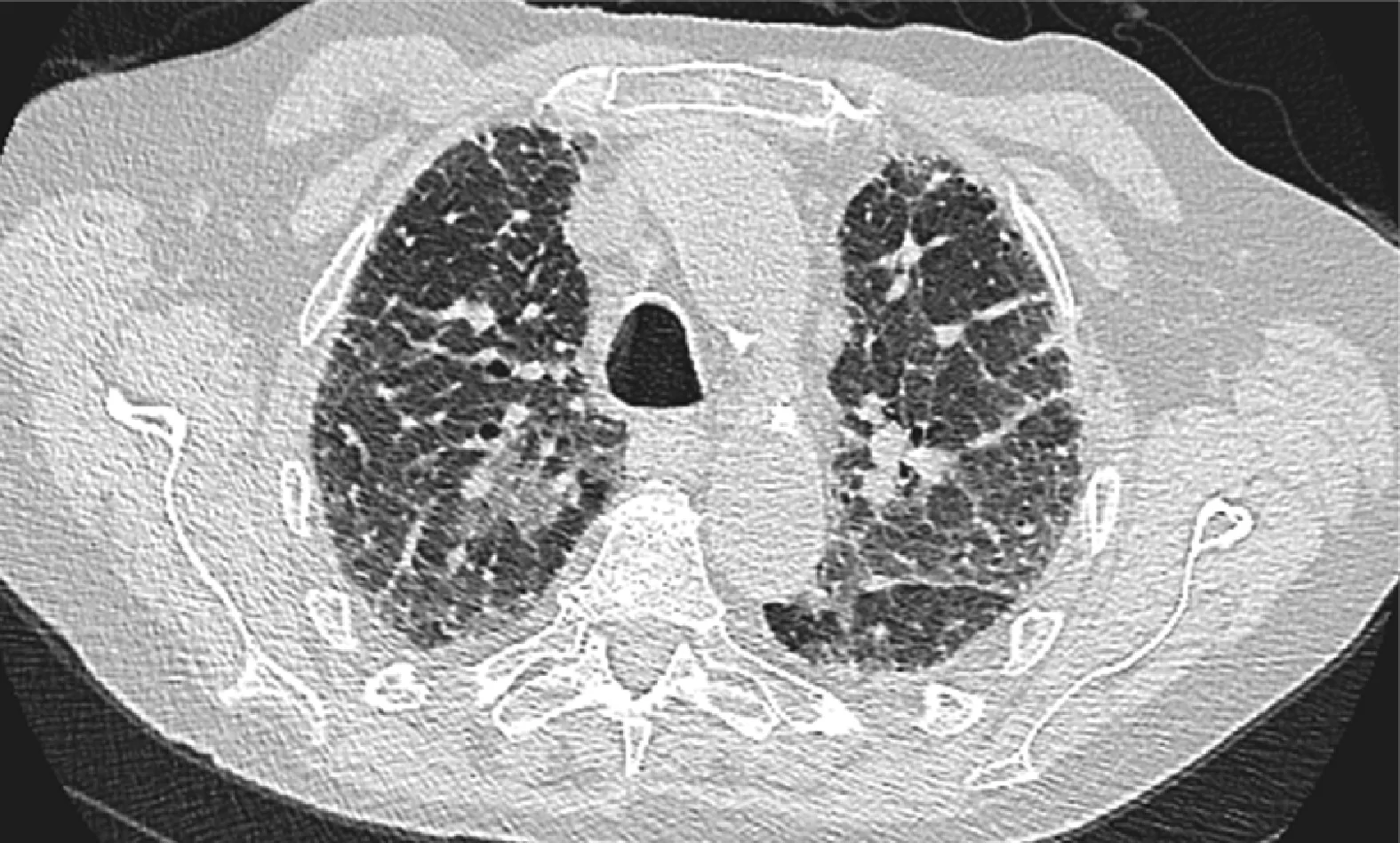
Patient 3 CT on admission for ILD exacerbation.

Case 4 Patient 4 is a 72 year old female with a history of atrial fibrillation, hyperthyroidism, hiatal hernia, obstructive sleep apnea, and unclassifiable interstitial lung disease. She was suspected to have either hypersensitivity pneumonitis based on feather exposure and water damage with mold in her home or Sjogrens based on prominent sicca symptoms and an antinuclear antibody titer of 1:640 homogenous; anti-ro and -la antibodies were negative and the patient declined minor salivary gland biopsy. Her CT was notable for fibrosis and 3 density sign (Fig. 4A and Fig. 4B).

**Fig. 4A:**
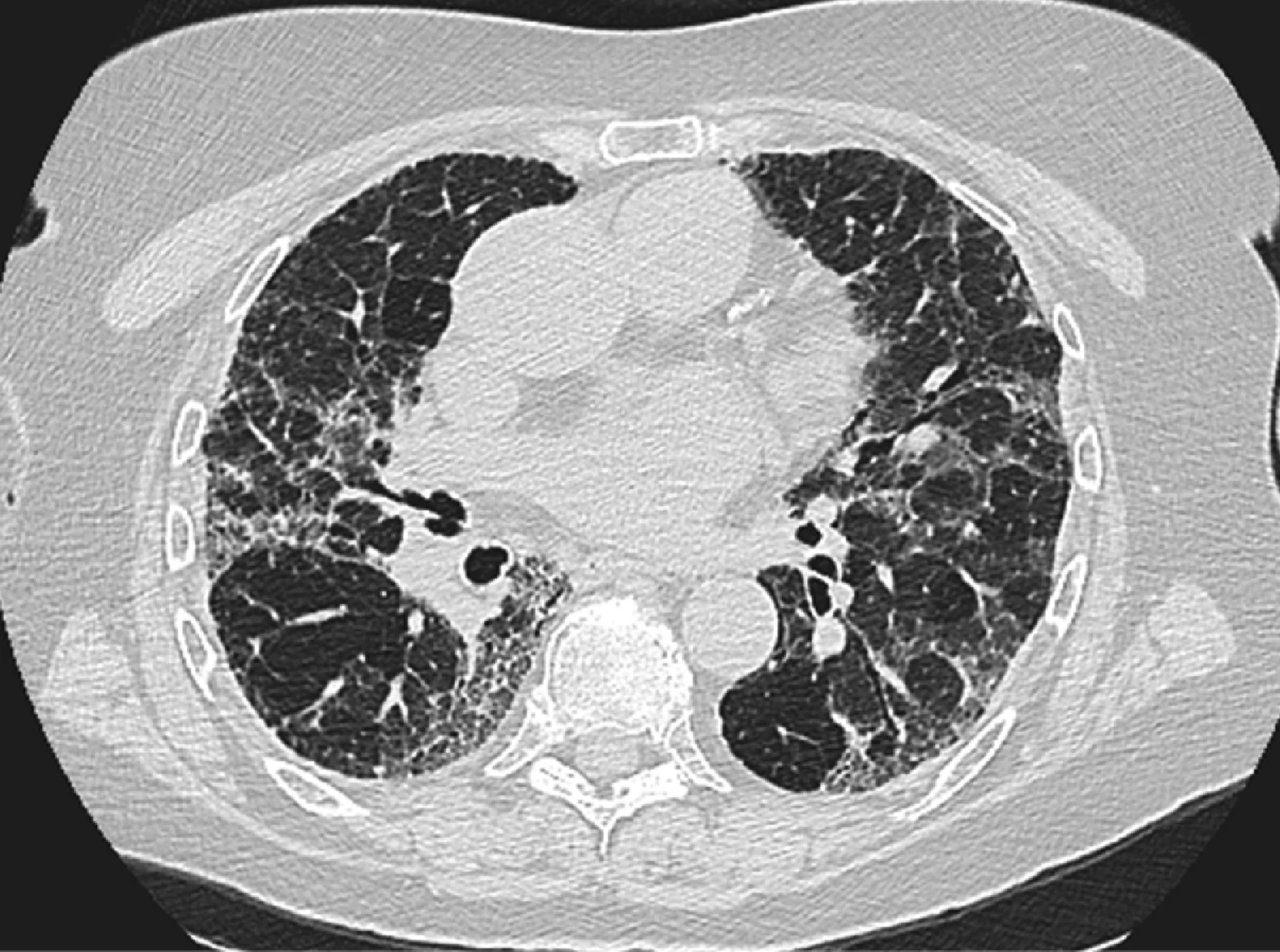
Patient 4 CT 1 year prior to initiation of nerandomilast.

**Fig. 4B:**
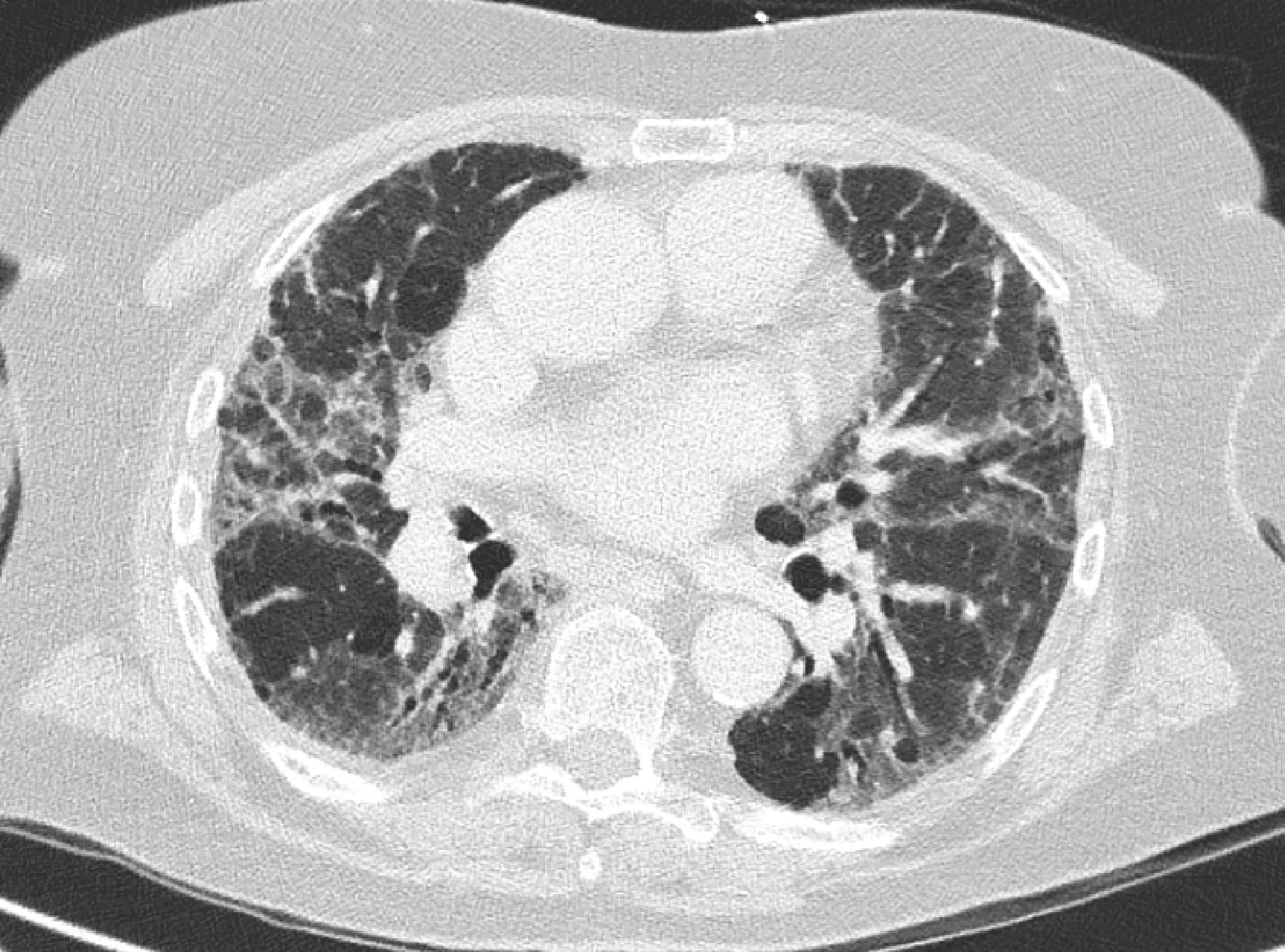
Patient 4 CT on admission for ILD exacerbation.

Due to worsening oxygen requirement and symptoms, she was initiated on nintedanib but was unable to tolerate it after just 1 dose and it was discontinued. Two months after stopping nintedanib she was initiated on nerandomilast 18mg twice daily. One week after starting nerandomilast, she developed cough and dyspnea. She was evaluated in UTSW pulmonary clinic 10 days after starting nerandomilast, and her forced vital capacity of 1.61L down from 1.82L 4 months prior. She was admitted to the hospital from pulmonary clinic. On admission, she was afebrile without leukocytosis, and on exam she had fine crackles in the lungs and no lower extremity edema. Respiratory viral panel was negative. CT showed increased ground glass opacities compared to 4 months prior. She has not yet been discharged from the hospital.

## 3 Patient 5

Patient 5 is a 74 year old male with PPF due to Sjogrens disease. The Sjogrens diagnosis is based on prominent sicca symptoms and SSA quantitative 32.6 (reference < 20). At his initial evaluation in the UTSW ILD clinic, he had a forced vital capacity of 34% predicted and an oxygen requirement of 15LPM with less than 1 minute of exertion, after which the walk test was terminated. He was initiated on nintedanib 150mg twice daily. He was declined for lung transplant and was referred to palliative care. Six months after beginning nintedanib he was initiated on nerandomilast 18mg twice daily. He began to feel ill with worsening cough and dyspnea 4 days after beginning to take nerandomilast. He called the clinic to report worsening exercise tolerance to less than 10 feet and increased oxygen requirement and was instructed to go to the emergency room. On admission, he was afebrile without leukocytosis. NT-pro BNP was 113, comparable to his baseline 6 months prior. CT revealed an increase in ground glass opacities (Fig. 5A, Fig. 5B). He was treated with systemic steroids and a taper. He self-discontinued nerandomilast. At his follow-up appointment 1 month after hospital discharge, he reported feeling better than at the time of admission but worse than 6 months prior. He did not have objective testing at his follow-up appointments due to his preference to avoid ongoing testing.

**Fig. 5A:**
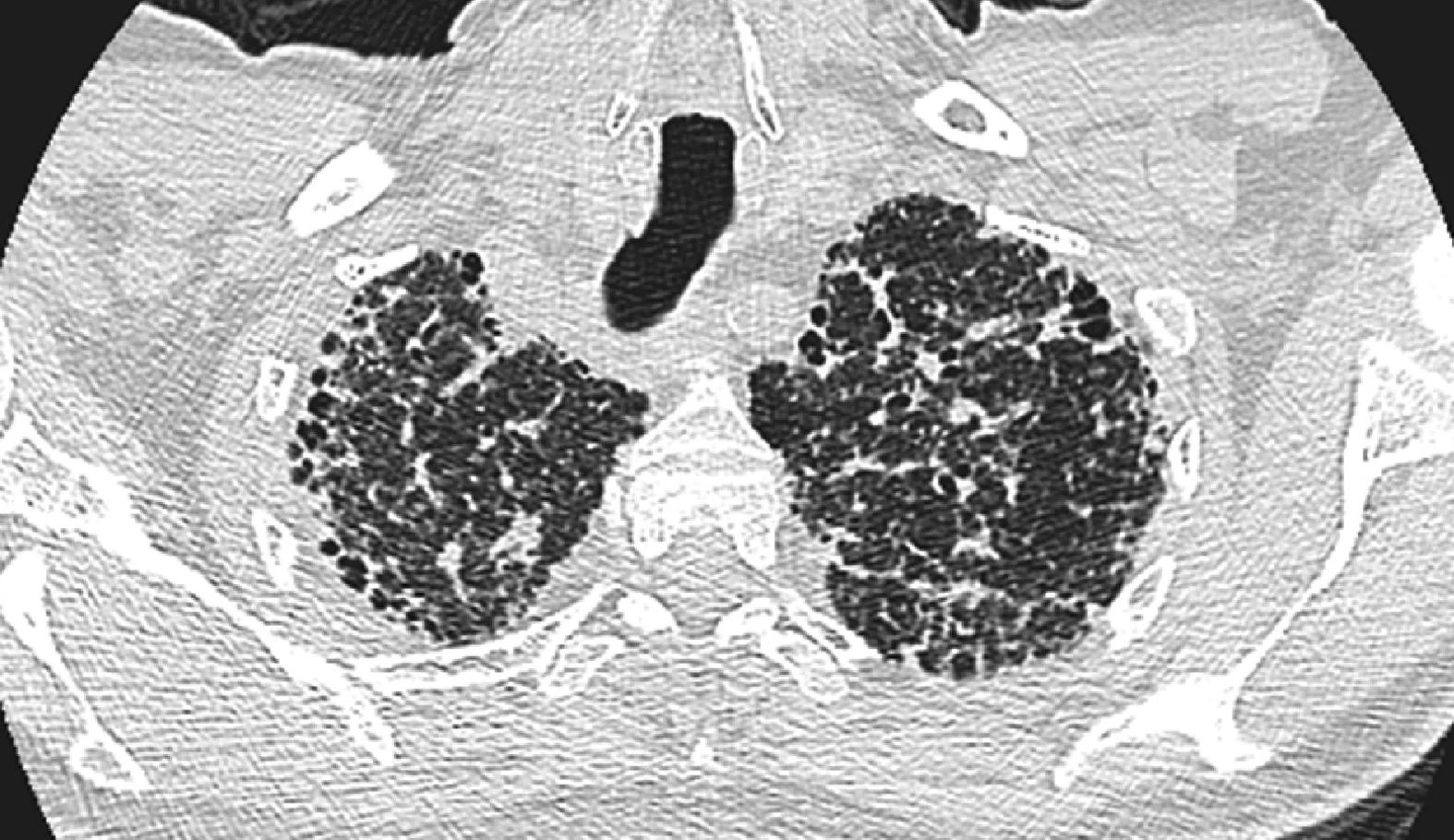
Patient 5 CT 6 months prior to initiation of nerandomilast.

**Fig. 5B:**
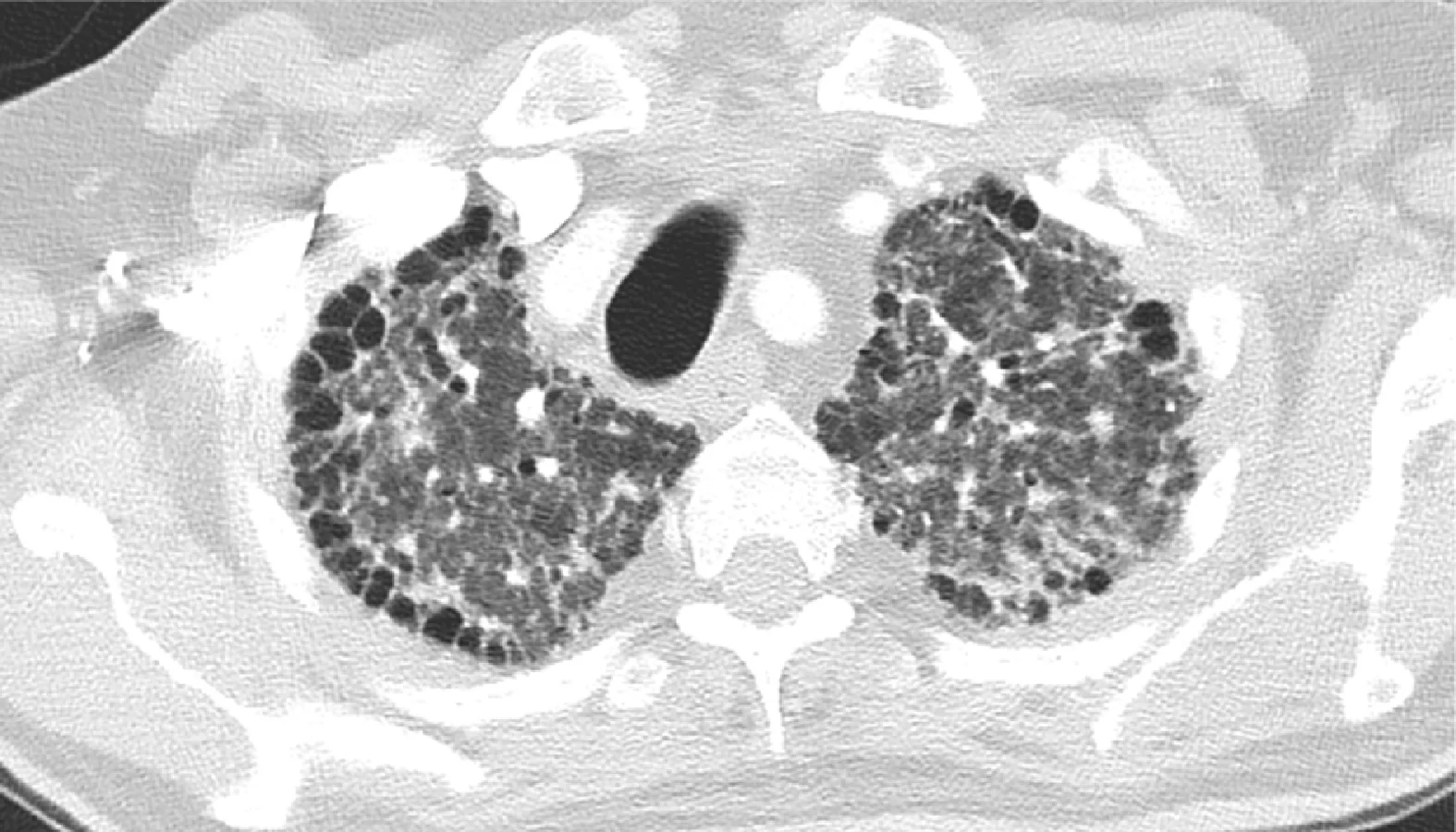
Patient 5 CT on admission for ILD exacerbation.

## 4 Discussion

This case series describes 5 patients with an acute exacerbation of interstitial lung disease within 1 month of initiation of nerandomilast. Notably, only one of these patients was on background therapy with nintedanib and none were on pirfenidone at the time of their exacerbation.

One possible mechanism for an increase in ILD exacerbations with initiation of nerandomilast is via upregulation of IL-17. Phosphodiesterase 4 (PDE4) interacts with cyclic nucleotide modulators to drive fibroblast proliferation, myofibroblast differentiation, and extracellular matrix synthesis [8,9]. PDE4 inhibition leads to an increase in intracellular cAMP levels, thus inhibiting lung fibroblast differentiation and proliferation [8,9]. cAMP-stimulating agents including cholera toxin, beta-adrenergic agonists, or adenosine receptor agonists that directly enhance IL-17A production in T cells can lead to Th17 cell differentiation and IL-17 secretion [[10], [11], [12]]. IL-17 overproduction, which can result from bacterial or viral infections, is a known driver of IPF exacerbations via neutrophil recruitment and activation of TGF-β signaling [13]. In a mouse model of acute exacerbation of IPF, increased proportions of IL-17^+^CD4^+^ have been described, and Th-17 gene knockout mice have milder exacerbations than wild type mice [13]. It is possible that nerandomilast could increase IL-17 production in the lung via cAMP, leading to increased risk of exacerbation.

Pirfenidone has been demonstrated to lower IL-17 levels [14], while nintedanib does not increase or decrease IL-17 in the lung [15], while decreasing IL-17 in alternate disease models including prostate cancer studies [16]. These findings may explain the occurrence of ILD exacerbations in patients who were not on background therapy at the time of initiation of nerandomilast. Further, because 43% of patients in the phase 3 trial of nerandomilast in PPF and 77% of patients in the phase 3 trial of nerandomilast in IPF were on background therapy [5,6], there may have been insufficient power to detect an increase in exacerbations in patients on nerandomilast monotherapy. However, the effect of pirfenidone and nintedanib on IL-17 has been demonstrated only in animal models, an observational study, and in vitro studies rather than in randomized clinical trials and thus their effect on IL-17 remains speculative [[14], [15], [16]].

Additional PDE-4 mediated immunologic effects include increasing anti-inflammatory IL-10 levels, reducing pro-inflammatory TNF-α and IL-23 [8,9]. Whether these cytokine can contribute to ILD exacerbations remains unclear.

Causation cannot be definitively demonstrated with a case series. Limitations are inherent in any case series, which include the absence of a control group, possible reporting bias, and inability to control for confounding factors such as heterogeneity of ILD diagnosis, baseline severity, comorbidities, infections, environmental triggers, and recent medication changes not reported by the patient that may have contributed to the outcome in these patients. In addition, because these patients were treated according to standard of care rather than a research protocol, BAL fluid sampling was not conducted at the time of exacerbation to evaluate IL-17 levels.

While acute exacerbations of ILD were not described in the clinical trials of nerandomilast, this case series indicates that further investigation of a potential side effect of nerandomilast, particularly as monotherapy, are warranted.

## Declaration of competing interest

The authors declare that they have no known competing financial interests or personal relationships that could have appeared to influence the work reported in this paper.
